# Acceptance of illness and satisfaction with life among malaria patients in rivers state, Nigeria

**DOI:** 10.1186/1472-6963-14-202

**Published:** 2014-05-03

**Authors:** Katarzyna Van Damme-Ostapowicz, Elżbieta Krajewska-Kułak, Paul JC Nwosu, Wojciech Kułak, Marek Sobolewski, Romuald Olszański

**Affiliations:** 1 Department of Integrated Medical Care, Medical University of Bialystok, Bialystok, Poland; 2 Madonna Institute of Tropical Medicine (Hygiene, Blood) and Pandemics, Madonna University, Elele, Nigeria; 3 Department of Pediatric Rehabilitation, Medical University of Bialystok, Bialystok, Poland; 4 Chair of Quantitative Methods, University of Technology in Rzeszów, Rzeszów, Poland; 5 Department of Maritime and Tropical Medicine, Military Medical Institute, Gdynia, Poland

**Keywords:** Acceptance of illness, Satisfaction with life, Malaria

## Abstract

**Background:**

Health condition is one of the basic factors affecting satisfaction with life, and the level of illness acceptance. The purpose of the study was to analyse the level of illness acceptance, the level of satisfaction with life among malaria patients, and the level of trust placed in the physician and the nurse.

**Methods:**

The study employs the method of diagnostic survey based on standardised AIS and SWLS scales, as well as Anderson and Dedrick’s PPTS and PNTS scales.

**Results:**

The average AIS level was 12 points, while the average level of SwL at the SWLS scale was 16.5 points. The average level of trust in the physician and the nurse amounted to 50.6 points and 51.4 points, respectively. The correlation between the level of illness acceptance and self-evaluated satisfaction with life was statistically significant, with R = 0.56. The marital status influenced the level of illness acceptance with p < 0.05 and the level of satisfaction with life with p < 0.05. The employment status affected the level of satisfaction with life with p < 0.05 and the level of illness acceptance with p < 0.05.

**Conclusions:**

The majority of malaria patients did not accept their illness, while the level of satisfaction with life was low. The majority of respondents trusted their physician and nurse. There is a statistically significant correlation between the level of illness acceptance and the self-evaluated satisfaction with life. The marital status had a statistically significant effect on the acceptance of illness and the satisfaction with life. The individuals who had a job demonstrated higher levels of quality of life and illness acceptance.

## Background

During the past decade, the occurrence of malaria and the related mortality rates have been reduced in all regions of the world, according to the World malaria report 2011. In 2010, there were an estimated 216 million cases of malaria in 106 endemic countries and territories in the world. An estimated 81% of these cases and 91% of deaths occurred in the WHO African Region [1]. Malaria is commonly considered to be a poverty-associated disease [2] and has been a major public health issue in many sub-Saharan African countries [3-5]. The disease has also been a major public health issue in Nigeria, a country with an estimated 170 million people [4,6]. Everyone living in all parts of Nigeria is at risk of malaria infection [4]. *Plasmodium falciparum* accounts for 90-95% of malaria infections in Nigeria. The transmission of malaria occurs throughout the year with the intensity higher in the southern parts of the country because of the longer rainy season that favours the breeding of mosquitoes [4]. The species of mosquitoes that commonly transmit malaria in Nigeria are *Anopheles gambiae, Anopheles funestus* and *Anopheles arabiensis *[4]. Malaria attacks are more frequent in young children aged below five years who lack the protective immunity, and are therefore more likely to suffer from severe malaria and to die from the disease. Severe malaria accounts for a third of all childhood deaths in Nigeria [4,6]. The WHO report states that for the 162 000 000 Nigerian residents “high transmission (≥1 case per 1000 of population)” is 100% [7].

The doctor-patient relationship is a complex one, determined by the actions and characteristics of participants, factors related to situation and environmental conditions [8]. A patient is usually an ill person, in a difficult situation [8]. The doctor-patient relationship is identical with the relationship of a doctor and a person in his or her illness. Illness usually changes the manner in which the surroundings are perceived – people and their behavior [8]. This is true for both family members, medical care providers and other patients [8,9].

With the progress of therapy and the improvement of the patient’s overall disposition, the patient may become able and willing to actively engage in contacts with the physician, and to participate in decisions regarding further therapy [8,10].

The quality of doctor-patient communication is a multidimensional concept which involves not only biomedical and psychosocial aspects of medical care, but also involves facets of the interaction itself. Moreover, fostering the doctor-patient relationship is considered an essential and universal value within medical practice [11].

Good Doctor-patient communication has a positive impact on patient satisfaction, adherence to treatment, health outcomes and well-being, and it has been linked to reduced anxiety, increased recall, and improved understanding [12]. Doctor-patient interaction, by individual roles and beliefs, and by a different understanding of health and well-being may be influenced by cultural and other factors not necessarily associated with the medical situation [12].

Literature on bioethics, in the evaluation of physician-patient relations, mentions a number of models of such relations. They include:

– A legalistic model,

– An economic or consumer model,

– A negotiated contract model,

– A religious model [13,14].

Concepts of quality of life (QoL) and satisfaction with life (SwL) are very useful for the processes of health enhancement, therapy and holistic care, and a rehabilitation process [15]. Researchers dealing with QoL and SwL, stress the fact that the evaluation should include the patient’s somatic condition, mental condition, social relations and physical fitness [16].

Health condition is one of the basic factors of high QoL and SwL [16], and the level of acceptance of the illness has a significant influence on the adaptation to the limitations imposed by the illness [17].

The level of acceptance of the illness has an important effect on the adaptation to the limitations imposed by the illness, dependence on others and evaluation of the patient’s own value [17]. The aforementioned determinants affect the subjective perception of QoL and SwL and determine the level of the patient’s own activity [17]. Any disease causes negative emotions, difficulties and imposes limitations or forces changes in performing social functions [18].

Specialist literature emphasises that that the higher the level of illness acceptance is, the better the patients adapt and the less intensive emotions they feel, which affects their evaluation of the quality of life [19].

The achievement of good results of therapy requires the professionalism of health-care workers on the one hand, and satisfaction with therapy – a subjective evaluation of the quality of medical service – on the other [20]. Quality of care and therapy is a level of consistence between the purpose of doctors’ and nurses’ actions and real care. Patient’s negative feelings may also be intensified by his/her poor health condition and the inability to understand the professional language used by health-care professionals [21]. Scientific literature stresses that the higher the acceptance of illness level, the better adaptation and lower intensity of negative emotions in patients, which affects their evaluation of quality of life [19].

Trust is an emotion showed to people, objects or institutions, including a company, government or society. We show trust towards a person who we believe will provide good advice having us, not himself/herself on his/her mind. Examples of public trust professions are physicians and nurses. A physician-patient trust constitutes a very important index of patient’s satisfaction, and therefore also the quality of medical services [22,23].

The quality of medical services is understood in various ways and defined in many different ways. It is defined differently by claimers, physicians and recipients of medical services – clients/patients [24]. Continuous progress, increasingly sophisticated medical techniques and equipment brought the necessity of discovering the patients’ needs and meeting those needs to the minds of decision-makers. Moreover, the effect of actions undertaken towards a particular patient is important – if a person being treated or contacting a healthcare system feels the expected benefits, he is satisfied, trusts the healthcare system, and trusts the physician who is taking care of him/her [25].

Although the impact of negative perceptions of access to effective malaria treatment is difficult to measure, treatment-seeking behavior has been shown to be strongly influenced by social relationships. People draw heavily on their social networks for advice; advice that is shaped by perceptions and rumors about health workers, quality of care and the health system in general [26]. Chuma [26] states further that, as Gilson notes, provider-patient relationships are influenced by patients’ attitudes towards providers [26]. For example, providers personally known to patients or of the same ethnic group or gender may be trusted more. However, providers may also introduce or reinforce negative patient perceptions through their own practices [26].

The determination of the level of illness acceptance broadly matches the more and more common interest of medicine studies in the issues related to the quality of life. This stems from the transformation observed in the ideology of medicine which recognises the need to assess the patient’s health holistically, including the description of the standard of living of the patient and the social status they enjoy in the environment in which they function. WHO defines the quality of life as individual’s perception of their position in life in the context of the culture and value systems in which they live and in relation to their goals, expectations, standards and concerns set by the features of their environment. As a source of tension, illness poses a challenge to the quality of life and satisfaction with it. Higher level of fear, anxiety and tension results from hindered adaptation to progressing illness. Although malaria affects many patients, the currently available literature lacks publications by other authors on the quality of life or the level of illness acceptance among patients suffering from this disease.

The aforementioned study analyses the level of malaria patients’ satisfaction with life in the context of their trust in the doctor/nurse and the level of acceptance of the disease.

In the opinion of authors the subject is justified both from the cognitive and practical point of view and is also found in the scope of health sciences.

At this point, it is worth noting that gaining good results from treatment involves the professionalism of healthcare professionals on the one hand, and the patients’ satisfaction with treatment – that is their subjective evaluation of the quality of healthcare services – on the other. It should be remembered, that nowadays, apart from involvement in making therapeutic decisions, patients expect medical services corresponding to the requirements of the current medical knowledge. The contemporary healthcare system has caused the patient to be transformed from a passive recipient of medical services into a party that evaluates and expresses opinions on the services rendered by a medical centre. A patient and his/her family, who play the role of a client, have the right to ask questions, choose and evaluate, and a service-renderer has to take his/her opinions into account. The quality of care and therapy is therefore a level of agreement between the purpose of doctors’ and nurses’ activities and an actual care. The patient’s negative feelings may be intensified by his/her poor health condition and an inability to understand the professional language used by healthcare professionals. Literature on the subject underlines that the higher the level of acceptance of a disease is, the better the adaptation and lower intensity of negative emotions in patients is.

Therefore, in the aforementioned evaluation, a very significant role is played by an objective evaluation of the conditions in which the patient is cared for, and by an analysis of doctor-patient or nurse-patient relations and the level of the patient’s acceptance of a disease.

We perceive the results obtained in this study as important in the achievement of the higher quality of patient care. We are convinced that they will be helpful in understanding the patient’s co-participation in therapeutic and nursing processes, and the patients’ expectations from medical staff, and therefore they will contribute to the improvement of the therapeutic process.

The advantage of our study is its uniqueness. The results of the study conducted among patients with malaria cannot be compared to studies conducted by other authors, because no studies have been conducted in this country so far using these methods.

Our current study, conducted in Nigeria on a group of 140 patients with malaria, was to demonstrate and diagnose life satisfaction of patients with malaria, diagnose the degree of acceptance of the disease and diagnose the degree of trust in the physician and nurses and to show any relationships between the studied parameters. Our study, which differs from another study conducted among 120 patients with malaria, in which we diagnosed the quality of life of patients with malaria, as well as satisfaction with life and acceptance of the disease of these patients, and we have analysed the relationships between the studied parameters.

## Methods

### Study area and population

The study was carried out in August 2010 among patients with confirmed malaria, diagnosed at the Madonna University Teaching Hospital in Elele, in South Nigeria, in the state of Rivers. Our study involved patients chosen at random, who came to the General Outpatient Department and who were diagnosed with malaria. The diagnosis of malaria in these patients was based on the presence of the development of one or more species of the Plasmodium genus in the microscopic examination of thick and thin blood smears stained using Giemsa stain at Madonna University Teaching Hospital in Elele.

The study lasted one month. The study was sample-based and the sample was selected at random. The following inclusion criteria were used in the study: patients admitted to the General Outpatient Department in Elele, patients with diagnosed malaria, patients between 15 and 65 years of age. The exclusions criteria included: persons not understanding the commands, illiterate persons, deaf persons, mentally ill persons, persons <15 years of age, persons >65 years of age.

Patients with a diagnosis of active parasitic invasion with a different etiology were excluded from the test group. Finally, the analysis included 140 patients, 69 women and 71 men, aged 15 to 65 years. The patients evaluated a physician and a nurse working permanently at the General Outpatient Department in Elele with whom the admitted patients had contact.

A doctor working for the General Outpatient Department (a hospital ward at which patients remain only for the time necessary to perform particular procedures, operating as an ambulatory, from an organizational point of view a department of the institution Madonna University Teaching Hospital in Elele) and a nurse working for the same department were subject to evaluation.

Madonna University Teaching Hospital in Elele accepted patients belonging to the Igbo tribe. Elele is a village with a population of 100,000. Madonna University Teaching Hospital in Elele has five departments, staffed by 35 doctors and 40 nurses. Between 30 and 60 patients visited the General Outpatient Department in Elele daily with a variety of ailments. The diagnosis of malaria in the patients was based on microscopic examination of thick and thin blood smears stained using Giemsa stain at Madonna University Teaching Hospital in Elele. The inhabitants of the Elele village come to the Madonna University Teaching Hospital in Elele where they receive medical assistance. Elele has 100,000 inhabitants and there are no other alternative clinics/hospitals in the area.

### Quantitative research methods

For the realisation of the accepted purposes a method of diagnostic survey was applied, using the following standardized scales:

– a standardized Satisfaction With Life Scale (SWLS) is a measure of life satisfaction [27].

Life satisfaction is one factor in the more general construction of subjective wellbeing. Life satisfaction can be assessed specific to a particular domain of life (e.g., work, family) or globally. The SWLS is a global measure of life satisfaction [27,28]. The SWLS consists of 5 items (In most ways my life is close to my ideal; The conditions of my life are excellent; I am satisfied with my life; So far I have achieved the important things I want in life; If I could live my life over, I would change almost nothing) that are completed by the individual whose life satisfaction is being measured. A respondent “agrees” or “disagrees” with statements using a 7-point gradation (1–7) of responses. Response score has a positive direction, which means that the higher the score, the higher the satisfaction with life.

This study utilised a standardized Anderson and Dedrick patient-physician trust scale, the reliability of which was confirmed by independent studies which were based on the concept that satisfaction associated with medical services, being a multi-factorial value, depends largely on the trust shown by the patient in the medical personnel taking care of them [29]. In this regard we were driven by a belief that satisfaction with a medical service composed of numerous factors depends largely on the trust placed by the patient in the healthcare personnel caring for him or her.

– an Anderson and Dedrick Patient-Physician Trust Scale (TPS), involving 11 issues (I doubt that my doctor really cares about me as a person; My doctor is usually considerate of my needs and puts them first; I trust my doctor so much that I always follow his/her advice; If my doctor tells me something is so, then it must be true; I sometimes distrust my doctor’s opinion; I trust my doctor’s judgments and opinions; I feel my doctor does not do everything he/she should about my medical care; I trust my doctor as to the way he/she treats my medical problems; My doctor is well qualified to treat medical problems; I can tell my doctor when he/she makes a mistake; I sometimes worry that my doctor may not keep my secrets) [29].

Respondents gave their answers according to a 5-grade scale: 1 – strongly disagree, 2 – disagree, 3 – neither yes, nor no, 4 – agree, and 5 – strongly agree.

– a Patient-Nurse Trust Scale (TNS), own modification based on the Anderson and Dedrick patient-physician scale, involving 11 issues (I doubt that my nurse really cares about me as a person; My nurse is usually considerate of my needs and puts them first; I trust my nurse so much that I always follow her advice; If my nurse tells me something is so, then it must be true; I sometimes distrust nurses; I trust nurses' judgments and opinions; I feel my nurse does not do everything she should about my medical care; I trust my nurse as to the way of my nursing; My nurse is well-qualified in nursing; I can tell my nurse when she makes a mistake; I sometimes worry that my nurse may not keep my secrets) [29]. Respondents gave their answers according to a 5-grade scale: 1 – strongly disagree, 2 – disagree, 3 – neither yes, nor no, 4 – agree, and 5 – strongly agree.

– AIS – standardized Acceptance of Illness Scale. The Acceptance of Illness Scale (AIS) consists of eight questions describing the consequences of poor health condition [30]. The questions regard the limitations imposed by the illness, lack of independence, the feeling of being dependent of others, and reduced self-esteem (I have problems with adapting to limitations imposed by my illness; I cannot do what I like best because of my health condition; My illness makes me sometimes feel unwanted; My health problems make me rely on others more than I want to; My illness makes me a burden for my family and friends; My health condition makes me feel a man of good value; I will never be self-dependent to the extent I would like to be; I think people around me feel often embarrassed because of my illness). Each question had a 5-grade scale of answers, and a respondent described their current health condition marking one suitable number: 1 – strongly agree, 2 – agree, 3 – don’t know, 4 – disagree, 5 – strongly disagree. A strong agreement means poor adaptation to the disease, and the lack of agreement – acceptance of the disease. The level of acceptance of the current health condition is measured by the sum of all points, within the range of 8 to 40 points. Three point ranges were created to define the level of acceptance. A score of 8 to 18 points corresponded to no acceptance of the disease, 19 to 29 – moderate acceptance, and 30 to 40 – good acceptance [28,30].

The response score has a positive direction, which means that the higher the score, the higher the acceptance of illness.

Moreover, the study used a patient survey questionnaire prepared especially for this research, which was not validated, the introduction of which contained: information on the purpose of the study, the voluntary character and anonymity of responses, the possibility of withdrawal from the study at any moment, regardless of the stage of the study, and information concerning the method of filling in questionnaires and scales.

The first part of the survey questionnaire also contained questions describing the demographics of the patient and the second part contained open questions regarding the patient’s health condition (medical history).

The survey questionnaires and scales were realised in English, which is the official language in Nigeria. They were filled in the presence of a Project Manager and a student of the last year of the Medicine Faculty at the Madonna University, who was trained in the purpose and assumptions of the study and who could speak the local language. The questionnaires were conducted and completed by the researcher. Before the beginning of the study, its purpose, assumptions and methods were carefully explained to each of respondents.

### Statistical analysis

Statistics was completed using the STATISTICA software.

Data were presented in the form of descriptive statistics, that is: arithmetic mean, median, maximum and minimum values, standard deviation (*s*), centile 25 and 75, scores of patients’ trust in the physician and in the nurse, the level of the acceptance of the illness (AIS) and satisfaction with life (SWLS). Distribution characteristics of the point values obtained are given for each scale, in the form of selected descriptive statistics. In addition, the distribution of values grouped into ranges is shown using histograms. If there are rules for the categorisation of point values for the given scale, the numerical and percentage structure of the scale transformed in this way is shown.

For the evaluation of the effect of the level of trust in medical personnel on the acceptance of the illness and satisfaction with life, a correlation analysis was performed, especially with Spearman rank correlation index.

The Mann–Whitney test (for two groups) and the Kruskal-Wallis test (for multiple groups) were used to investigate the diversity of trust in the medical staff, acceptance of the disease and satisfaction with life based on gender and then based on the age of the respondents, as well as to assess the statistical significance of the differences in the scales between the compared groups.

Additionally, the differentiation of trust in medical personnel, acceptance of illness and satisfaction with life in relation to gender, and to the age of surveyed subjects was analyzed. The Mann–Whitney test (for two groups) and the Kruskal-Wallis test (for multiple groups) were used for the evaluation of the statistical significance of the differences of levels of scales between compared groups. The distribution of AIS and SWLS values in individual age groups was illustrated in box and whiskers plots.

For the SWLS scale, the results were also presented in another form, divided into categories of quality of life and acceptance of illness, using a chi squared independence test for drawing statistical conclusions, and the results were presented in an adjective scale.

The distribution of the SWLS, TPS, TNS and AIS scales has been described in the summary table. For these four tested parameters, the skewness coefficient (A) was tested and the result of the Shapiro-Wilk normality test (ps-W) determined.

The rationale for the selection of non-parametric tests and the non-parametric correlation coefficient as a tool for dependency analysis and statistical inference is the significant deviation from the normal distribution and a fairly large asymmetry in the distribution of individual scales. The results used are presented in Table 1.

**Table 1:** Distribution of SWLS, TPS, TNS and AIS scores described with selected descriptive statistics and results of Shapiro-Wilk normality test

**Scales**	$\overset{¯}{x}$	**Me**	***s***	**min**	**max**	***A***	***p***_**S-W**_
SWLS	16,5	16,0	3,1	7	23	-0,32	0,0053**
TPS	50,6	51,0	3,8	43	55	-0,34	0,0000***
TNS	51,4	52,0	3,5	43	55	-0,69	0,0000***
AIS	12,0	10,5	4,0	8	26	1,08	0,0000***

### Ethical considerations

The study received consent from the Madonna University Ethics Committee in Elele Decision no. 15/E/2010 and Ethical Committee of the Medical University in Białystok no. R-I-002/518/2010.

Individual interviews were only started after the purpose of the study had been clearly explained to the participant and an informed consent form was obtained.

## Results

One hundred and forty malaria patients participated in the study, including 49% of females and 51% of males. Among the surveyed participants, less than half (46%) were married.

The age distribution was as follows: 15–18 years - 5%, 19–30 years- 32%, 31–50 years- 52% and over 50 years - 11%.

All of the participants were living in rural areas. The majority of respondents (67%) – nearly two thirds of them – were unemployed. Their hospitalization (during the study) was, in the majority of cases, a repeated hospitalization (for over 90% of participants).

### Satisfaction with life scale (SWLS)

**Figure 1:**
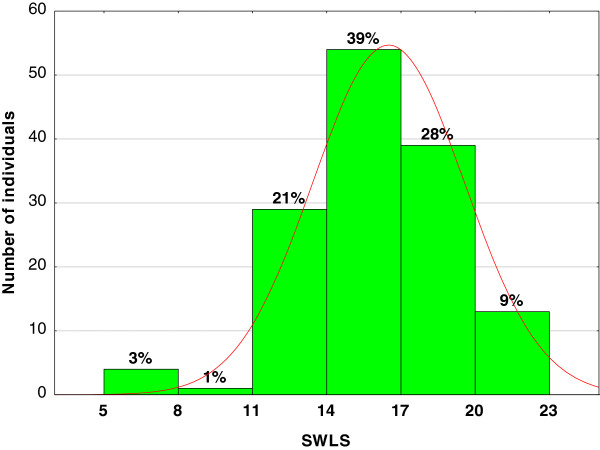
Scores in the SWLS scale.

About a third (37%) of the respondents scored above 17 points while the rest scored less than half the maximum score on the SWLS (Figure 1).

### Tests for the level of trust and acceptance of illness

#### ***The patient-physician trust scale***

The mean score from the patient - physician trust score was 50.6 (S.D + 3.8) and details are shown in Table 2.

**Table 2:** Patient-physician trust score

**Characterictic**	$\overset{¯}{x}$	**Me**	***s***	**min**	**max**
Trust in the physician (TPS)	50.6	51.0	3.8	43	55

#### *The patient – nurse trust scale*

Trust in the nurse was even higher. At least 25% of the surveyed demonstrated trust at the highest possible level (55 points), which is reflected by the value of centile 75.

The average level of trust in the nurse was 51.4 points; the minimum value for that scale was 43, and the maximum 55, with a standard deviation of 3.5, *Me* was 52.0.

A significant majority of participants trusted their nurse, which is reflected by high scores in the TPS scale. 1% of respondents fit into the score range of 40–43 points, 10% of respondents fit into the score range of 43–46 points, 16% of respondents fit into the score range of 46–49 points, 27% of respondents fit into the score range of 49–52 points, 46% of respondents fit into the score range of 52–55 points.

### Acceptance of illness scale

The acceptance of illness demonstrated by participants was very low – at a mean level of 12 points, with one in two persons on the level of 10 points or less, one in every four persons – 9 points or less, with a standard deviation of 4.0, *Me* was 10.5, *c*_75_ = 15.0, minimum = 8, maximum = 26.

The majority of participants did not accept their illness, which is also reflected by low scores in the AIS scale. 65% of respondents fit into the score range of 8–12 points, 19% of respondents fit into the score range of 12–16 points, 19% of respondents fit into the score range of 16–20 points, 2% of respondents fit into the score range of 20–24 points, 1% of respondents fit into the score range of 24–28 points.

Following the grouping of AIS scores and the transformation into an adjective scale, it is evident that almost all responses (94%) fell into the category of “no acceptance for illness” (Table 3).

**Table 3:** Determination of respondents’ level of acceptance of illness

**Level of illness acceptance**	**Count**	**Percent**
None	131	93.6%
Moderate	9	6.4%

The overall results for these four scales SWLS, TPS, TNS, AIS are summarised in the summary Table 1, which also contains the value of the skewness coefficient *(A)* and the result of the Shapiro-Wilk normality test (*p*_S-W_). All of the analysed scales show statistically significant derogation from the normal distribution. The SWLS, TPS and TNS scales have a left-sided asymmetry and the AIS scale a right-sided asymmetry. These results also provide a rationale for the selection of non-parametric tests as tools of statistical inference. However, since the asymmetry in the distribution of the entire population studied does not have to be the same in each group, which differ by age, marital status, and employment status, the tables in group breakdowns include both the mean and the median, so that in this way the reader can self-assess the direction and strength of asymmetry.

The mean level of Acceptance of Illness Scale was 12 points, Me was 10.5, the standard deviation was 4.0, the minimum value for the scale was 8, and the maximum value was maximum 26, the value of the skewness coefficient (*A*) was -1,08 and the result of the Shapiro-Wilk normality test (*p*_S-W_) was 0,0000***.

The mean level of SwL in the SWLS scale was 16.5 points, Me was 16.0, the standard deviation was 3.1, the minimum value for the scale was 7, and the maximum value was 23, the value of the skewness coefficient (*A*) was -0,32 and the result of the Shapiro-Wilk normality test (*p*_S-W_) was 0,0053**.

The average level of trust in the physician was 50.6 points, Me was 51.0, the standard deviation was 3.8, the minimum value for the scale was 43, and the maximum value was 55, the value of the skewness coefficient (*A*) was -0,34 and the result of the Shapiro-Wilk normality test (*p*_S-W_) was 0,0000***.

The average level of trust in the nurse was 51.4 points; Me was 52.0, the standard deviation was 3.5, the minimum value for that scale was 43, and the maximum 55, the value of the skewness coefficient (*A*) was -0,69 and the result of the Shapiro-Wilk normality test (*p*_S-W_) was 0,0000***(Table 1).

### Correlations between selected scales

#### *Acceptance of illness and satisfaction with life*

**Figure 2:**
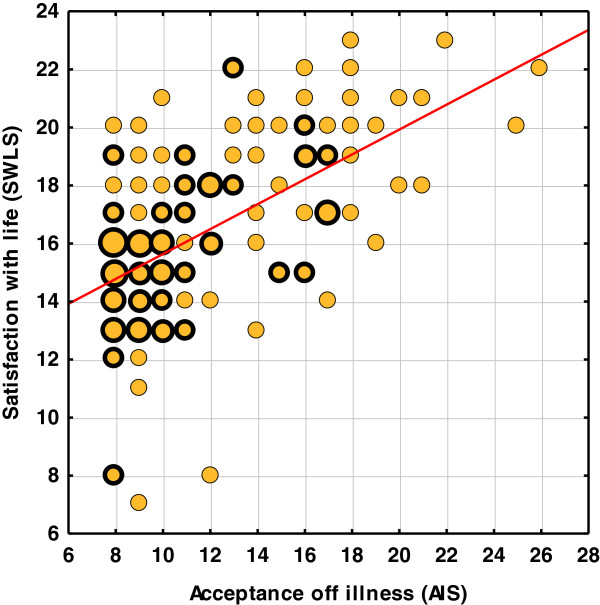
A correlation between the level of acceptance of illness and self-evaluated satisfaction with life.

There is a statistically significant, moderately powerful, correlation between the level of the acceptance of the illness and self-evaluated SwL (SWLS), with R = 0,56 (p = 0,0000***) (Table 4). The higher the acceptance of the illness, the higher the satisfaction with life (Figure 2).

**Table 4:** A correlation between the level of acceptance of illness and self-evaluated satisfaction with life

**Acceptance of illness**	**Satisfaction with life (SWLS)**
AIS	0.56 (*p* = 0,0000***)

#### ***Trust in the physician and trust in the nurse***

**Figure 3:**
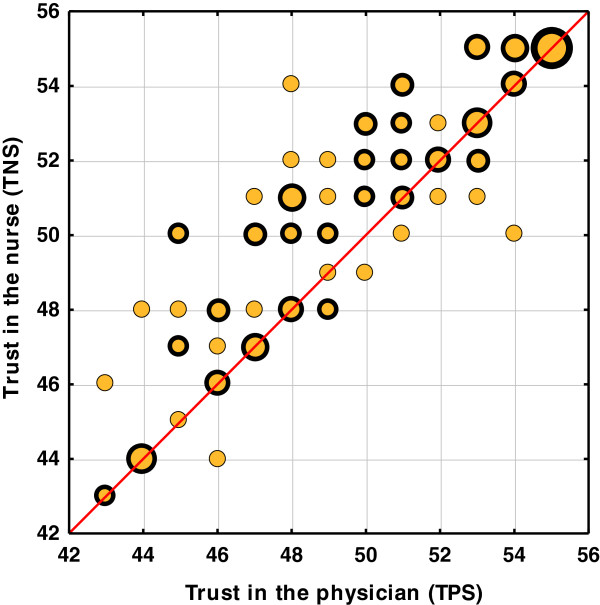
A correlation between the level of trust in the physician and trust in the nurse.

The analysed correlation between trust in the physician and trust in the nurse demonstrated that the correlation between those values is *R* = 0.93. It is therefore very strong, which is supported by the graphic representation in Figure 3. Participants who score their trust in the physician highly, usually have a higher trust in the nurse as well.

### Demographic

Gender differentiates only the level of trust in physicians, which is significantly higher (by approx. 2 points in the TPS scale) in females, with p = 0.0195*, the arithmetic mean was 51.3, *Me* was 53.0.

On the other hand, age clearly differentiates the level of the acceptance of the illness, being markedly higher in the group 15–18 years (by as much as approx. 20 points, on average) and in the group 19–30 years (by approx. 14 points on average), compared to just over 10 points in both older age groups, with p = 0.0000***. A similar effect is exerted by age on the evaluation of SwL, p = 0.0016**, dropping in older age groups.

**Figure 4:**
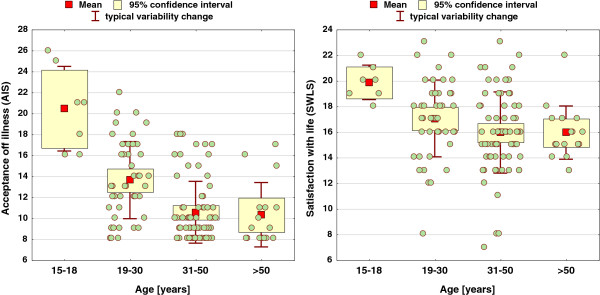
Distribution of AIS and SWLS scores in individual age groups.

The distribution of AIS and SWLS values in individual age groups is shown in box and whisker plots (Figure 4).

Marital status differentiates the level of acceptance of illness (AIS score), where p = 0.0000*** and quality of life SWLS, w p = 0.0263* (Table 5).

**Table 5:** Effect of the factor of marital status on the value of measurement scales

**Measurement scale**			**Marital status**				***p***
	**Married**		**Single**		**Widow/widower**		
	$\overset{¯}{x}$	**Me**	$\overset{¯}{x}$	**Me**	$\overset{¯}{x}$	**Me**	
Trust in doctor (TPS)	50.4	50.5	50.2	51.0	52.5	55.0	0.0520
Trust in nurse (TNS)	51.2	52.0	51.1	51.0	52.9	55.0	0.1077
**AIS**	**10.9**	**10.0**	**13.9**	**13.0**	**9.4**	**9.0**	0.0000***
**SWLS**	**16.6**	**16.0**	**16.8**	**18.0**	**15.1**	**15.0**	0.0263*

However, differences are smaller than in the case of analysis of the age factor and are probably a consequence of age differences between the compared groups –the majority of single individuals were between 19 and 30 years old, and the majority of married ones were between 31 and 50 years old.

Marital status has an impact on the level of acceptance of the disease and single persons are characterised by a higher level of acceptance of the disease.

Professional status is a very strong factor affecting trust in medical personnel: trust in doctor (TPS), where p = 0,0152* and trust in nurse (TNS), where p = 0,0038**, and especially the level of satisfaction with life (SWLS), where p = 0.0000*** and the level of acceptance of illness (AIS), where p = 0.0000*** (Table 6).

**Table 6:** Effect of the professional status factor on the value of measuring scales

**Measuring scales**	**Professional status**				***p***
	**Employed (*N*** **= 44)**		**Unemployed (*N*** **= 94)**		
	$\overset{¯}{x}$	**Me**	$\overset{¯}{x}$	**Me**	
Trust in doctor (TPS)	49.6	49.5	51.0	53.0	0,0152*
Trust in nurse (TNS)	50.4	50.5	51.8	53.0	0,0038**
AIS	14.5	14.5	10.9	10.0	0.0000***
SWLS	18.1	18.0	15.8	16.0	0.0000***

However, the nature of the collected data does not allow for a detailed analysis of the impact of employment status on quality of life and attitudes towards the disease. The group of employed patients was dominated by sellers, and other professions were practised mostly by 1–2 people, which precludes any reliable comparison.

Important and worth noting, is the fact that the gender, age and marital status structure is very similar in groups of employed and unemployed subjects. Therefore, it is not an apparent correlation, but the actual effect of professional status on attitude to the disease.

Those people, however, have a slightly lower trust in doctor/nurse, but in this case, the score difference between both groups is, although statistically significant, not very large.

For the SWLS scale, the results were also presented in another form, with division into categories of quality of life and acceptance of disease, using the chi-squared independence statistical test making statistical conclusions (Table 7). As can be seen, also following transformation into the adjective scale, there are some statistically significant differences in the distribution of satisfaction with life – in favor of the employed group, where p = 0.0000***, in which there are relatively more “satisfied” (11) and “neutral” (6) individuals, and clearly less “highly unsatisfied” ones (2).

**Table 7:** Effect of professional status on the SWLS scale

**SWLS adjective scale**	**Professional status (*p*** = 0.0000***)		**Total**
	**Employed**	**Unemployed**	
Highly unsatisfied	1 (2.3%_↓_)	2 (2.1%_↓_)	3
Very unsatisfied	2 (4.5%_↓_)	27 (28.7%_↓_)	29
Rather unsatisfied	24 (54.5%_↓_)	58 (61.7%_↓_)	82
Neutral	6 (13.6%_↓_)	5 (5.3%_↓_)	11
Rather satisfied	11 (25.0%_↓_)	2 (2.1%_↓_)	13
Total	44	94	138

Other social and demographic factors analyzed in our study demonstrated no differentiation.

## Discussion

In professional journals there are no reports on the problem of the evaluation of satisfaction with life and the evaluation of level of acceptance of illness by malaria patients. We believe that the results of this study are important for improving the quality of patient care. We are confident they will be helpful in the understanding of the patient’s cooperation in the therapeutic and nursing process, as well as of the patients’ expectations from medical professionals. Thus they will account for the perfection of the therapeutic process.

The presented paper may very well act as a basis for planning activities aimed at the sensitization of healthcare professionals on the discussed problem and the optimization of the professional approach of medical personnel to a malaria patient.

Therefore it seems purposeful to realise studies that would allow the evaluation of the malaria patients’ satisfaction with life and evaluation of the level of acceptance of the illness.

This study demonstrated that the majority of malaria patients did not accept their illness. The respondents’ assessment of satisfaction with life was low and there was a statistically significant correlation between: trust in healthcare provider, the level of acceptance of illness and self-evaluation of satisfaction with life. Our studies show that marital status has an impact on the level of acceptance of the disease and single persons are characterised by a higher level of acceptance of the disease.

Employed individuals demonstrated a higher quality of life and a better acceptance of the illness. The study demonstrated that the majority of respondents place trust in their doctor and nurse.

### Satisfaction with life scale (SWLS)

This study demonstrated that the mean level of SwL in the SWLS scale was rather low.

A moderate dissatisfaction with life, where the mean evaluation of satisfaction with life in the SWLS scale, 17.7 points, dominates in the study population in the research conducted by Van Damme-Ostapowicz et al., carried out among malaria patients in Nigeria [28]. The similarity of the results may be due to a similar methodology of the studies conducted.

The studies show the results of the satisfaction with life of patients suffering from malaria and can help to improve the quality of life of the infected by drawing the attention of physicians and nurses to the important issue of quality of life.

### Acceptance of illness scale (AIS)

This study demonstrated that the respondents’ acceptance of the illness was low. The majority of participants did not accept their illness, which was reflected by low scores in AIS scale.

Similar results were obtained by authors of the study carried out among malaria patients in Nigeria [28], which showed that the majority of participants did not accept their illness. The category of “no acceptance” had a dominating share in the adjective scale, that category was chosen by the majority of respondents.

According to the literature, people who accept their illness are people who understand the disease and are conscious of its course, who, at the same time, demonstrate an optimistic and hopeful attitude to life, trust physicians, trust therapeutic methods and actively participate in therapy [31]. However, one can observe a discrepancy between the results of this study and data provided in the literature in case of patients who do not accept their illness which is presumably a cause of burden in their lives even if they fully trust their healthcare provider.

According to literature, the higher the degree of illness acceptance is, the better the patients adjust and the less severe negative emotions they experience.

Studies on the knowledge of malaria and behaviour associated with the disease, completed in various parts of the world - Africa [32-35], Asia [36] and Europe [37] demonstrate a similar, wrong attitude to malaria. Even in Nigeria, Oregba *et al.*[38], a survey carried out among respondents in a society occupying the south-western part of the country, demonstrated that the level of knowledge of the inhabitants was very low, and the observed level is a result of myths and erroneous beliefs sustained by caregivers and parents [38]. People’s ability to submit themselves to medical procedures and to the therapeutic process depends on the level of acceptance of those procedures, and on the level of understanding of the nature of the disease [39].

Studies performed by Oladele and Kauna [40], studies by Atkinson et al. [41] and studies by Kaliyaperumal and Kumera [42] have demonstrated that knowledge associated with action/management in the case of malaria was significantly correlated with the level of education and other important cultural, social and economic factors.

Quality of care and treatment is the level of consistency between the purpose of the work of physicians and nurses, and the actual care. Patients’ negative feelings may also be compounded by their poor health and not being familiar with the specialised language spoken by health professionals. According to literature, the higher the degree of acceptance of the disease, the better the adjustment and less severe the negative emotions are in patients. The studies show the results of the acceptance of the disease by malaria patients, which can help to improve the level of quality of care for the patients, and will be helpful in understanding the patient’s co-participation in the process of healing and care, as well as the expectations of patients from the medical staff, which will contribute to the improvement of the therapeutic process.

### The patient-physician trust scale

The presented results demonstrated that the level of trust in the physician was very high – as the average level of trust in the physician was 50.6 points.

In the study by Krajewska-Kułak et al. [43], regarding the evaluation of trust placed by patients in their doctors in relation to the country of residence – Poland or Belarus, - there was no doubt as to the proper care for the patient by the doctor for 53.3% of residents of Poland and 56% of residents of Belarus, while 83.3% of patients from Poland and 56% of patients from Belarus stated that a doctor usually considers patients’ needs and values them highly [43].

A high level of trust in the physician and full compliance were also declared by 90% of respondents in the study completed by Chilicka et al. on a group of 150 female in-patients of gynaecological-obstetrics wards in Poland [44].

The authors of the current study, however, expected a difference in the assessment of physician trust in malaria patients, compared with the trust to the physician in gynecological and obstetric patients. The similarity of the results may be due to a similar methodology of the studies conducted.

Whereas studies by Krajewska-Kułak et al. performed on a group of 109 female in-patients of gynaecological-obstetric wards in Greece demonstrated that patients in Greece had a lower trust in their doctors compared to patients in Poland, and more often declared that a doctor did not do his/her best for their care [45].

### The patient – nurse trust scale

Our own studies demonstrated that trust in the nurse was even higher, because at least 25% of respondents demonstrated the highest possible level of trust (55 points), and the average level of trust in the nurse was 51.4 points.

The results of the study conducted among patients with malaria cannot be compared to studies conducted by other authors, because no studies have been conducted in this country so far using these methods.

The studies showing the results of the assessment of malaria patients’ trust in the physician or nurse can help to improve the level of the quality of care for the patients, and will be helpful in understanding the patient’s co-participation in the process of healing and care, as well as the expectations of patients from the medical staff, which will contribute to the improvement of the therapeutic process.

### Correlations between selected scales

#### ***Trust in personnel and acceptance of illness and satisfaction with life***

An analysis of the effect of the level of trust in medical personnel on the acceptance of illness and SwL demonstrated that there was a statistically significant correlation between trust in the physician and trust in the nurse, although the power of the correlation is very low.

The character of the correlation is somewhat surprising – a negative sign of the correlation coefficient says that people demonstrating a higher trust in the physician/nurse have a lower acceptance of the illness and have a lower SwL. However, it should be underlined that the correlation is very weak, and the reduction of acceptance of illness and SwL in patients who trust doctors/nurses more is minimal compared to the rest of participants.

As such, perhaps, the negative direction of the correlation between the level of trust in the provider and quality of life (SWLS) corresponds to an obscure effect of the disease advancement level. People more severely ill present lower SWLS scores, and, on the other hand, may present higher trust in providers, as a result of more frequent contact with doctors and nurses.

**Figure 5:**
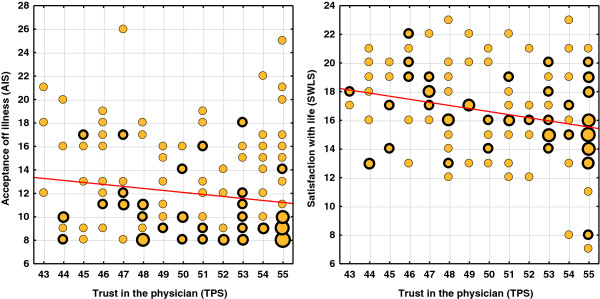
A correlation between trust in the physician and acceptance of illness and satisfaction with life.

As the assessment of trust in the physician and trust in a nurse were at an almost identical level for the majority of those taking part in the survey, graphs show only one of those values associated with AIS and SWLS (Figure 5).

#### ***Trust in personnel and acceptance of illness and satisfaction with life***

An analysis of the effect of the level of trust in medical personnel on the acceptance of illness and satisfaction with life demonstrated that there is a statistically significant correlation between trust in the physician and the nurse, although the strength of that correlation was very low.

The results of the study conducted among patients with malaria cannot be compared to studies conducted by other authors, because no studies have been conducted in this country so far using these methods.

#### ***Acceptance of illness and satisfaction with life***

As this study has demonstrated, there is a statistically significant, moderately powerful, correlation between the level of the acceptance of the illness and self-evaluated SwL, indicating that the higher the acceptance of the illness, the higher SwL.

In the study of malaria patients in Nigeria [28], the authors demonstrated that there is a statistically significant correlation between the level of illness acceptance and the quality of life and satisfaction with life, and the plus sign of the correlation coefficient substantiates the statement that the higher level of illness acceptance determines the higher quality of life.

They demonstrated that the correlation between acceptance of illness and satisfaction with life was 0.40*** and that it was a very highly statistically significant correlation with a low correlation power [28].

#### ***Trust in the physician and trust in the nurse***

Our own study demonstrated that people who have a high trust in doctors, also usually have a higher trust in nurses. As demonstrated by the results of the study by Schoenfelder et al. [46] on 8428 patients, aimed at the identification of the determinants of patient satisfaction, there are 10 determinants of global patient satisfaction, and the outcome of therapy was generally the strongest prognostic factor, followed by the politeness of nurses. While elements reflecting information about treatment had no significant effect on patient satisfaction [46].

As a study completed in Kenya [26] demonstrated, the fact that the negative perceptions towards health workers’ age, gender and qualifications were expressed mainly by elderly women implies that they may have considerable cultural concerns, firstly, in exposing their bodies for injection to providers who were predominantly young males, and secondly, in tolerating the language of providers, which they regard as disrespectful to their age and social status as married women [26].

### Demographic

The nature of the collected data does not allow for a detailed analysis of the impact of employment status on quality of life and attitudes towards the disease. The group of employed patients was dominated by sellers, and other professions were practised mostly by 1–2 people, which precludes any reliable comparison.

Our own studies demonstrated that gender has a slight differentiating effect only on the level of trust in the physician, which is characteristically higher - 2 points in the TPS scale – in females.

The results of the study conducted among patients with malaria cannot be compared to studies conducted by other authors, because no studies have been conducted in this country so far using these methods.

An analysis of the results obtained in research conducted by Van Damme-Ostapowicz et al. [28], carried out among malaria patients in Nigeria demonstrated that men are characterized by a higher acceptance of illness. Moreover, the study demonstrated the existence of a statistically significant correlation between the level of acceptance of illness and satisfaction with life [28].

Our study demonstrated that age clearly differentiates the level of the acceptance of the illness, because participants belonging to younger age groups showed a higher acceptance of the illness, compared to older patients. A similar effect of age was found for SwL, the evaluation of which is reduced in older age groups.

Studies completed in Nigeria by Iwalokun et al. proved that the use of western medicines was associated with having a younger age, while self-medication was favourably practised by the male gender [47].

The present study demonstrated that marital status differentiated the level of acceptance of illness and satisfaction with life. Marital status has an impact on the level of acceptance of the disease and single persons are characterised by a higher level of acceptance of the disease.

The results obtained in our study demonstrated that professional status was a factor which very strongly affected trust in medical personnel: trust in doctor and trust in nurse, and particularly the level of satisfaction with life and the level of acceptance of the illness. Employed participants had a higher satisfaction with life, which was also visible following transformation to the adjective scale, where there were some statistically significant differences in the distribution of satisfaction with life – in favor of the employed group; and employed subjects also demonstrated a higher acceptance of their illness.

The nature of the collected data does not allow for a detailed analysis of the impact of employment status on quality of life and attitudes towards the disease. The group of employed patients was dominated by sellers, and other professions were practised mostly by 1–2 people, which precludes any reliable comparison.

However, studies by Dillip et al. in Tanzania demonstrated that being a farmer was associated with a delay in malaria treatment [48].

### Limitations

It is clear that we must note the limitations of the present study.

• Extent of generalizability of their results:

Patients studied, the number of which was not large.

One place where the patients were examined and treated.

• Pre-testing of the survey questionnaire and effects on validity of the tools:

Preliminary tests were not carried out because we were guided by the fact that the SWLS, AIS, TPS and TNS scales are scales which have been used many times in the past, while the rest of the questionnaire contained questions about sex, age and employment.

• Translation of questionnaire into the local language:

The authors did not make a translation into local language and it was not adapted to the culture, because the survey questionnaires and scales were realised in English, which is the official language in Nigeria. They were filled in the presence of a Project Manager and a student of the last year of the Medicine Faculty at the Madonna University, who was trained in the purpose and assumptions of the study and who could speak the local language. The questionnaires were conducted and completed by the researcher. Before the beginning of the study, its purpose, assumptions and methods were carefully explained to each of respondents.

• The authors did not discuss the impact of malaria severity with complications on the acceptance of the disease and satisfaction with life. The authors did not classify the severity of the disease (severity of malaria). Such an analysis was not performed.

Analysis of the impact of malaria severity including complications on the acceptance of the disease and satisfaction with life was not carried out.

## Conclusions

The study demonstrated that the majority of malaria patients did not accept their illness. The respondents’ assessment of satisfaction with life was low. The majority of respondents trust their physician and nurse. There was a statistically significant correlation between: the level of acceptance of the illness, self-evaluation of satisfaction with life and trust in a healthcare provider. Marital status had a statistically significant effect on the acceptance of the illness and satisfaction with life. Our studies show that marital status has an impact on the level of acceptance of the disease and single persons are characterised by a higher level of acceptance of the disease. Employed individuals demonstrated a higher quality of life and a better acceptance of the illness.

### Recommendation

We consider the results of this study as essential in order to improve the level of quality of care for patients. We are confident that they will be helpful in understanding the patient’s co-participation in the treatment and care process as well as the expectations of patients from the medical staff, thus contributing to the improvement of the therapeutic process. At this point it is worth noting that the achievement of good results of treatment requires both the professional approach of health care workers, and also satisfaction from the treatment, that is a subjective assessment of the quality of medical services. It should be remembered that in this day and age the patient, in addition to participating in decisions about their care, also expects medical services from the provider that correspond to the needs of current medical knowledge. The studies can help to improve the quality of life of the infected by drawing the attention of physicians and nurses to the important issue of quality of life. In the future it would be desirable to carry out similar studies in other parts of Nigeria.

## Abbreviations

WHO: World health organization; QoL: Quality of life; SwL: Satisfaction with life; TPS: Patient-physician trust scale; TNS: Patient-nurse trust scale; AIS: Acceptance of illness scale; SWLS: Satisfaction with life scale; p: Test probability also called a critical significance level; 3.0: Arithmetic mean; Me: Median, middle value; max: Maximum value; min: Minimum value; s: Standard deviation; c25: Centile 25; c75: Centile 75; R: Correlation; p: Points.

## Competing interests

The authors declare that they have no competing interests.

## Authors’ contributions

All the authors were involved in the design of the study. KVDO was responsible for the implementation of the study in the field and the draft of the manuscript. EKK conceived the study, and participated in its design and coordination and supported the drafting of the manuscript; NP JC participated and supported in the drafting of the manuscript; WK coded the data and supervised data entry; MS coded the data, supervised data entry and analyzed the study data; RO analyzed the study data. All the authors were involved in finalizing the manuscript, read and approved the final version.

## Pre-publication history

The pre-publication history for this paper can be accessed here:

http://www.biomedcentral.com/1472-6963/14/202/prepub

## References

[B1] WHO World Malaria Report 2011 [http://www.who.int/mediacentre/news/releases/2011/malaria_report_20111213/en/index.html] website last accessed 04 Sept 2012

[B2] De Castro MC Fisher MG Is malaria illness among young children a cause or a consequence of low socioeconomic status? evidence from the United Republic of Tanzania Malar J 2012 14 161 10.1186/1475-2875-11-161 22571516 PMC3439375

[B3] Yewhalaw D Kassahun W Woldemichael K Tushune K Sudaker M Kaba D Duchateau L Van Bortel W Speybroeck N The influence of the Gilgel-Gibe hydroelectric dam in Ethiopia on caregivers’ knowledge, perceptions and health-seeking behaviour towards childhood malaria Malar J 2010 14 47 10.1186/1475-2875-9-47 20146830 PMC2829593

[B4] Arogundade ED Adebayo SB Anyanti J Nwokolo E Ladipo O Ankomah A Meremikwu MM Relationship between care-givers’ misconceptions and non-use of ITNs by under-five Nigerian children Malar J 2011 14 170 10.1186/1475-2875-10-170 21696622 PMC3146898

[B5] De-Graft Aikins A Arhinful DK Pitchforth E Ogedegbe G Allotey P Agyemang C Establishing and sustaining research partnerships in Africa: a case study of the UK-Africa academic partnership on chronic disease Global Health 2012 14 29 10.1186/1744-8603-8-29 22897937 PMC3475042

[B6] Meet the health workers at the frontlines of disease control: Q&A with a rural health workerhttp://www.malariaconsortium.org/news-centre/nigeria-malaria-control-in-the-world-s-most-endemic-country.htm. website last accessed 08 July 2013

[B7] World Health Organization (WHO) WORLD MALARIA REPORT 2013 [http://www.who.int/malaria/publications/country-profiles/profile_nga_en.pdf] website last accessed 02 July 2013

[B8] Henszen-Niejodek I Jarosz M Warszawa JM Patient-physician relations; diagnostics and therapeutic set Medical psychology 1988 PZWL 301 331

[B9] Buetow S Fuehrer A Macfarlane K Daniel McConnell D Moir F Huggard P Doerr H Development and validation of a patient measure of doctor-patient caring Patient Educ Couns 2012 14 264 269 10.1016/j.pec.2011.04.007 21592717

[B10] Adams R Price K Tucker G Nguyen AM Wilson D The doctor and the patient—How is a clinical encounter perceived? Patient Educ Couns 2012 14 127 133 10.1016/j.pec.2011.04.002 21890301

[B11] Butalid L Verhaak PF Boeije HR Bensing JM Patients’ views on changes in doctor-patient communication between 1982 and 2001: a mixed-methods study BMC Fam Pract 2012 14 80 10.1186/1471-2296-13-80 22873783 PMC3460773

[B12] Mehnert A Lehmann C Koch U Doctor-patient interaction: dealing with difficult situations Bundesgesundheitsblatt Gesundheitsforschung Gesundheitsschutz 2012 14 1134 1143 10.1007/s00103-012-1544-x 22936481

[B13] Emanuel EJ Emanuel LL Four models of the physician-patient relationship JAMA 1992 14 2221 2226 10.1001/jama.1992.03480160079038 1556799

[B14] Pellegrino ED Thomasma DC The virtues in medical practice 1993 New York: Oxford University Press

[B15] Raphael D Rootman I, Goodstadt M, Hyndman B, McQueen DV, Potvin L, Springett J, Ziglio E Evaluation of quality - of - life initiatives in health promotion. In Evaluation in health promotion Principles and perspectives 2001 Toronto: Centre for Health Promotion at the University of Toronto 123 127

[B16] Addington-Hall J Kalra L Measuring quality of life: Who should measure quality of life? BMJ 2001 14 1417 1420 10.1136/bmj.322.7299.1417 11397754 PMC1120479

[B17] Al Robaee AA Alzolibani AA Narrowband ultraviolet B phototherapy improves the quality of life in patients with psoriasis Saudi Med J 2011 14 603 606 21666943

[B18] Kułak W Kondzior D Acceptance of chronic low back pain in actively working patients Prog Health Sci 2011 14 81 88

[B19] Das A Ravindran TS Factors affecting treatment-seeking for febrile illness in a malaria endemic block in Boudh district, Orissa, India: policy implications for malaria control Malar J 2010 14 377 10.1186/1475-2875-9-377 21192825 PMC3224374

[B20] Ping L Alain J A three-model comparison of the relationship between quality, satisfaction and loyalty: an empirical study of the Chinese healthcare system Health Serv Res 2012 14 436 10.1186/1472-6963-12-436 PMC3520735 23198824

[B21] Chou SM Chen TF Woodard B Yen MF Using SERVQUAL to evaluate disconfirmation of nursing service in Taiwan J Nurs Res 2005 14 75 83 10.1097/01.JNR.0000387529.22642.2b 15986309

[B22] Plomp H Ballast N Trust and Vulnerability in doctor-patient relations in occupational health Occup Med 2010 14 261 269 10.1093/occmed/kqq067 20511267

[B23] Kahama-Maro J D’Acremont V Mtasiwa D Genton B Lengeler C Low quality of routine microscopy for malaria at different levels of the health system in Dar es Salaam Malar J 2011 14 332 10.1186/1475-2875-10-332 22047131 PMC3217957

[B24] Nabbuye-Sekandi J Makumbi FE Kasangaki A Kizza IB Tugumisirize J Nshimye E Mbabali S Peters DH Patient satisfaction with services in outpatient clinics at Mulago hospital, Uganda Int J Qual Health Care 2011 14 516 523 25 10.1093/intqhc/mzr040 21775313

[B25] Liabsuetrakul T Petmanee P Sanguanchua S Oumudee N Health system responsiveness for delivery care in Southern Thailand Int J Qual Health Care 2012 14 169 175 10.1093/intqhc/mzr085 22215759

[B26] Chuma J Okungu V Molyneux C Barriers to prompt and effective malaria treatment among the poorest population in Kenya Malar J 2010 14 144 10.1186/1475-2875-9-144 20507555 PMC2892503

[B27] Diener E Emmons RA Larson RJ Griffin S Juczyński Z SWLS-satisfaction with life scale Measurement Tools in Promotion and Health Psychology 2001 Warsaw: Laboratory of Psychological Tests of Polish Psychological Association 168 172

[B28] Van Damme-Ostapowicz K Krajewska-Kułak E Rozwadowska E Nahorski WL Olszański R Quality of life and satisfaction with life of malaria patients in the context of acceptance of the disease: quantitative studies Malar J 2012 14 171 10.1186/1475-2875-11-171 22616635 PMC3386889

[B29] Anderson LA Dedrick RF Development of the in physician scale: a measure to assess inter-personal trust in patient-physician relationships Psychol Rep 1990 14 1091 1100 10.2466/pr0.1990.67.3f.1091 2084735

[B30] Felton BJ Revenson TA Hinrichsen GA Juczyński Z AIS- acceptance of illness scale Measurement tools in Promotion and Health Psychology 2001 Warsaw: Laboratory of Psychological Tests of Polish Psychological Association 158 167

[B31] Lewko J Polityńska B Kochanowicz J Zarzycki W Okruszko A Sierakowska M Jankowiak B Górska M Krajewska-Kułak E Kowalczuk K Quality of life and its relationship to the degree of illness acceptance in patients with diabetes and peripheral diabetic neuropathy Adv Med Sci 2007 14 Suppl 1 144 146 18229653

[B32] De La Cruz N Crookston B Dearden K Gray B Ivins N Alder S Davis R Who sleeps under bednets in Ghana? A doer/non-doer analysis of malaria prevention behaviours Malar J 2006 14 61 10.1186/1475-2875-5-61 16867194 PMC1553454

[B33] Govere J Durrheim D La Grange K Mabuza A Booman M Community knowledge and perceptions about malaria and practices influencing malaria control in Mpumalanga Province, South Africa S Afr Med J 2000 14 611 616 10918892

[B34] Tilaye T Deressa W Community perceptions and practices about urban malaria prevention and control in Gondar Town, northwest Ethiopia Ethiop Med J 2007 14 343 351 18326344

[B35] Ouattara AF Raso G Edi CVA Utzinger J Tanner M Dagnogo M Koudou BG Malaria knowledge and long-lasting insecticidal net use in rural communities of central Côte d’Ivoire Malar J 2011 14 288 10.1186/1475-2875-10-288 21970433 PMC3196930

[B36] Simsek Z Kurcer MA Malaria: knowledge and behaviour in an endemic rural area of Turkey Public Health 2005 14 202 208 10.1016/j.puhe.2004.03.011 15661131

[B37] Van Damme-Ostapowicz K Krajewska-Kułak E Olszański R Nahorski W Problems involving contagious diseases and tropical medicine: New challenges for health care staff Adv Clin Exp Med 2011 14 461 471

[B38] Oreagba AI Onaajole AT Olayemi SO Mabadeje AFB Knowledge of malaria amongst caregivers of young children in rural and urban communities in southwest Nigeria Trop J Pharmaceut Res 2004 14 299 304

[B39] Adongo PB Kirkwood B Kendall C How local community knowledge about malaria affects insecticide-treated net use in Northern Ghana Trop Med Int Health 2005 14 366 378 10.1111/j.1365-3156.2005.01361.x 15807801

[B40] Akogun OB John KK Illness-related practices for the management of childhood malaria among the Bwatiye people of north-eastern Nigeria Malar J 2005 14 13 10.1186/1475-2875-4-13 15723706 PMC553996

[B41] Atkinson JAM Fitzgerald L Toaliu H Taleo G Tynan A Whittaker M Ian R Andrew V Community participation for malaria elimination in Tafea Province, Vanuatu: Part I. Maintaining motivation for prevention practices in the context of a disappearing disease Malar J 2010 14 93 10.1186/1475-2875-9-93 20380748 PMC2873527

[B42] Kaliyaperumal K Kumera A Knowledge and health seeking behavior for malaria among the local inhabitants in an endemic area of Ethiopia: implications for control Health 2010 14 575 581

[B43] Krajewska-Kułak E Wróblewska K Kruszewa R Szpakow A Kułak W Baranowska A Jankowiak B Krajewska K Kondzior D Lewko J Łukaszuk C Rolka H Klimaszewska K Kowalczuk K Sierakowska M Szyszko-Perłowska A Van Damme-Ostapowicz K Jaszewski M Kowalewska B Chilińska J Gołębiewska A Evaluation of patient-physician trust with Anderson and Dedrick scale Probl Hig Epidemiol 2008 14 414 418

[B44] Chilicka M Krajewska-Kułak E Rozwadowska E Terlikowski R Hładuński M Młynarczyk I Assessment of physician-patient relationships at the obstetrics and gynecology departments Nurs 21st C 2010 14 25 29

[B45] Krajewska-Kułak E Chilicka M Kułak W Adraniotis J Chadzopulu A Rozwadowska E Assessment of physician-patient trust in the obstetrics and gynecology departments in Poland and Greece Gynecol Pol 2011 14 905 910 22384626

[B46] Schoenfelder T Klewer J Kugler J Determinants of patient satisfaction: a study among 39 hospitals in an in-patient setting in Germany Int J Qual Health Care 2011 14 503 509 10.1093/intqhc/mzr038 21715557

[B47] Iwalokun BA Agomo PU Egbuna KN Iwalokun SO Adebodun V Olukosi OO Aina O Okoh HI Agomo CU Ajibaye O Orok O Enya VNV Akindele S Akinyele MO Environmental Survey and Health Seeking Behavior of Caregivers of Children Suspected of having Malaria in Takwa-Bay, Lagos State J Med Med Sci 2011 14 675 687

[B48] Dillip A Hetzel MW Gosoniu D Kessy F Lengeler C Mayumana I Mshana C Mshinda H Schulze A Makemba A Pfeiffer C Weiss MG Obrist B Socio-cultural factors explaining timely and appropriate use of health facilities for degedege in south-eastern Tanzania Malar J 2009 14 144 10.1186/1475-2875-8-144 19563640 PMC2712476

